# Tremor Research Group Essential Tremor Rating Scale (TETRAS): Assessing Impact of Different Item Instructions and Procedures

**DOI:** 10.5334/tohm.64

**Published:** 2020-10-05

**Authors:** William G. Ondo, Belen Pascual, On behalf of the Tremor Research Group

**Affiliations:** 1 Methodist Neurological Institute, Houston, TX, US; 2 Weill Cornell Medical School, US

**Keywords:** essential tremor, scales, TETRAS

## Abstract

**Introduction:**

The Tremor Research Group Essential Tremor Rating Scale (TETRAS) is a well-validated instrument to assess essential tremor. However, similar to all other tremor rating scales, specific instructions for individual tasks are based mostly on expert opinion and tradition. Several tasks have multiple possible variations that have never been compared to determine if they impact score.

**Methods:**

Using blinded, randomized videotapes, a group of tremor experts evaluated multiple ET patients to determine: 1. whether assessments of spirals and writing samples are similar if the rater only sees the end result as opposed to actually watching the task, 2. whether arm tremor ratings (postural and wing-beating) are similar if the subjects hold both hands out concurrently vs. if they only hold one arm out at a time, 3. whether heal to shin tremor scores vary between supine and sitting, and 4. compared cursive vs script writing samples.

**Results:**

Intraclass correlation coefficients (ICC) were excellent (>0.95) for all arm assessments. Writing tremor was rated worse if only rating the spiral/writing photos (p < 0.05) rather than also viewing the writing process, arm tremor scores were higher if each arm was rated individually (p < 0.001), heal to shin scores were higher when done sitting (p = 0.01), and cursive writing tended to be rated higher than script (p = 0.08).

**Discussion:**

Minor procedure differences when administering the TETRAS can significantly alter results.

The Tremor Research Group Essential Tremor Rating Scale (TETRAS) was developed by the Tremor Research Group (TRG) as a reliable, quick and easy tool to assess essential tremor [1,2,3,4]. The scale is well validated and sensitive to therapeutic intervention [1], however, similar to all other tremor rating scales, specific instructions for individual tasks are based mostly on expert opinion and tradition [2]. To our knowledge, actual comparisons of different specific instructions to evaluate tremor have never been attempted. Several tasks have multiple possible variations that have never been compared to determine if they impact score, i.e. should postural tremor be done one arm at a time or both raised concurrently etc. The amount of task homogeneity required to not impact score is entirely unknown. We therefore compared different ways of performing portions of the TETRAS to see how subtle differences in scale administration might impact scores.

## Methods

We assessed different ways to evaluate four major components of the TETRAS: 1. whether assessments of spirals and writing samples are similar if the rater only sees the end result on a photograph/paper as opposed to actually watching the task on video and seeing the end result, 2. whether arm tremor ratings (postural and wing-beating) are similar if the subjects holds both hands out concurrently vs. if they only hold one arm out at a time, 3. we compared different ways to assess leg tremor (sitting heal to shin vs. supine heal to shin), 4. we compared cursive vs. printed writing samples.

Videotapes (12 subjects) and writing samples (24 different subjects) were done at Methodist Neurological Institute (WO) after subjects signed video consent (Methodist Research Institute IRB). Videos were all done in front of the same background. Sixteen movement disorders specialists from the TRG met and scored the 12 ET subjects performing the first 3 tasks. At a later date, 15 TRG members blindly rated cursive and script writing samples from 24 different subjects. The mean scores of the 16 and 15 member groups were then used to statistically analyzed the different methods.

Specifically, for comparison of “live” writing vs. photographed writing, scores of spirals and handwriting samples were compared when done while watching a video of task. Approximately 2 months later, randomized, blinded, still photographs of those same spirals and drawings were distributed and scored by the same TRG members for comparison.

Leg tremor scores were done two different ways. Heel-knee-shin task was performed while the patient was supine and again while the subject was sitting. Edited video segments showing these tasks were then randomized prior to assessments, but the order was modifed manually if needed so that no two compared segments from the same subject were seen within 6 video segments of each other, as part of the 36 total video sequence.

Both postural and wing beating tremor was rated in each arm when the arms were concurrently extended versus when each arm was extended individually. Again, video segment order was randomized among all 48 total videos.

The writing samples were collected at a later date from 24 ET subjects (WO). Subjects were randomized to write “This is a sample of my best handwriting” in cursive then print, or print then cursive. Writing samples were cut, coded and randomized (N = 48 samples total), then rated at another meeting by 15 Tremor Research Group members.

Paired samples *t* tests were conducted to assess whether there were differences between the six major components of the TETRAS: 1. arm tremor unilateral vs bilateral, 2. wing tremor unilateral vs bilateral, 3. heel to shin supine vs sitting, 4. handwriting on photo vs video, 5. Spirals on photo vs video, and 6. Cursive vs Scrip. Normality assumption was tested for each paired samples *t* test. The size of the effect was calculated using the Cohen’s *d*. We followed the interpretation suggested by Cohen, *d* = 0.2 was considered a ‘small’ effect size, 0.5 was considered a ‘medium’ effect size and 0.8 a ‘large’ effect size. Pearson correlations between each two paired tests were also calculated. To determine the internal consistency among raters, intraclass correlation coefficients (ICCs) were calculated for each method of the six components of TETRAS using a 2-way mixed model and an absolute agreement definition. Statistical analyses were performed with SPSS (version 20).

## Results

**Figure 1:**
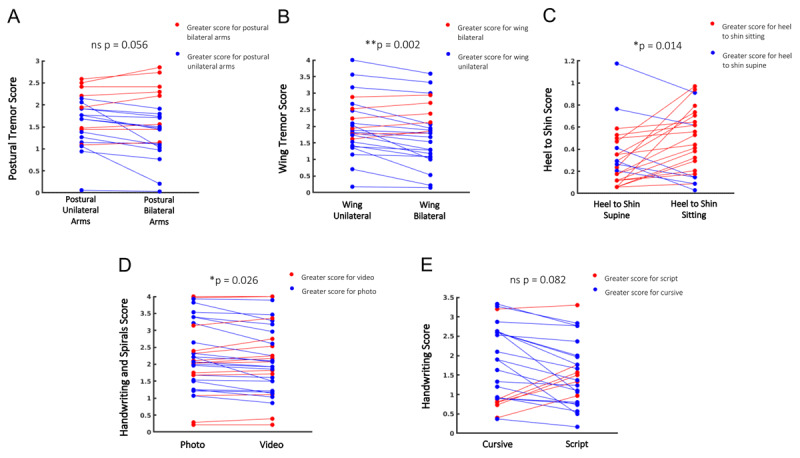
Mean scores (N = 15 raters in A-D and N = 16 raters in E) comparison of individual tremor subjects. Blue shows lower score in second measure, red denotes higher score in second measure. **A.** Postural tremor done individually vs. done concurrently, **B.** Wing-Beating tremor done individually vs. done concurrently, **C.** Heal to shin done while supine vs. while done sitting, **D.** Combined handwriting and spiral scores when rated only by a photograph vs. when video was also viewed of the subject performing the task, **E.** Comparison of cursive writing vs. script writing.

Normality assumption was met for all Paired samples *t* tests calculated in this study. As expected, there was generally a strong correlation between the two different methods assessed for the tasks. The means and standard deviations for the major assessed components of the TETRAS are presented in Table 1 and Figure 1.

**Table 1:** Comparison of different methods for six components of the TETRAS.

TETRAS components	Mean *(S.D.)* Method 1	Mean *(S.D.)* Method 2	Paired measures *t* test (*p* value)	Paired Samples Correlation	Size Effect *d*

Postural tremor Unilateral vs. bilateral	Unilat 1.655 (0.594)	Bilat 1.519 (*0.724*)	*t* (21) = 2.022 *p* = 0.056	*r* = 0.903 *p* < 0.001	0.43
Wing-beating Unilateral vs. bilateral	Unilat 1.996 (*0.862*)	Bilat 1.766 (*0.912*)	*t* (23) = 3.521 *p* = 0.002	*r* = 0.936 *p* < 0.001	0.72
Tremor Postural Wing Unilateral vs. bilateral	Unilat 1.833 (*0.757*)	Bilat 1.648 (*0.828*)	*t* (45) = 3.948 *p* < 0.001	*r* = 0.923 *p* < 0.001	0.58
Heel to shin Supine vs. sitting	Supine 0.310 (*0.267*)	Sitting 0.461 (*0.290*)	*t* (23) = 2.651 *p* = 0.014	*r* = 0.502 *p* = 0.013	0.54
Handwriting (photo vs. video)	Photo 1.938 (*1.113*)	Live video 1.827 (*1.169*)	*t* (11) = 1.452 *p* = 0.174	*r* = 0.974 *p* < 0.001	0.42
Spirals (photo vs. video)	Photo 2.482 (*1.008*)	Live Video 2.402 (*0.955*)	*t* (23) = 1.784 *p* = 0.088	*r* = 0.976 *p* < 0.001	0.36
Spirals & handwriting (photo vs. video)	Photo 2.301 (*1.065*)	Live Video 2.210 (*1.051*)	*t* (35) = 2.333 *p* = 0.026	*r* = 0.976 *p* < 0.001	0.39
Cursive vs. Script	Cursive 1.768 (*0.976*)	Script 1.536 (*0.824*)	*t* (23) = 1.819 *p* = 0.082	*r* = 0.772 *p* < 0.001	0.37

Postural/wing-beating unilateral arm assessments were scored higher when viewed individually compared to when assessed with both arms raised concurrently (p < 0.001). The difference was more pronounced in the wing-beating position (Table 1).

Heal to shin done while sitting was scored higher compared to when done in the supine position (p = 0.01), and correlation between the two methods was low, r = 0.5.

Combined spiral and writing samples were scored higher when viewed only as a photo, than when rated while watching the subjects perform the tasks (p < 0.05) but correlation was very high, r > 0.9.

Cursive writing trended to be rated as more severe than script (p = 0.08) and correlation was moderately high, r = 0.77.

Intraclass correlation coefficients (ICC) were greater than 0.95 for all methods of the six components of TETRAS except for heel to shin supine and sitting (Table 2). ICC was general very similar between the compared tasks.

**Table 2:** Intraclass Correlation Coefficient (ICC) of different methods for six components of the TETRAS.

TETRAS components	ICC	Lower Bound	Upper Bound

Postural tremor Unilateral	0.983	0.971	0.992
Postural tremor Bilateral	0.982	0.970	0.991
Wing-beating Unilateral	0.992	0.986	0.996
Wing-beating Bilateral	0.989	0.980	0.995
Heel to shin Supine	0.801	0.666	0.899
Heel to shin Sitting	0.797	0.661	0.897
Cursive	0.963	0.935	0.982
Script	0.956	0.921	0.979
Spiral	0.966	0.908	0.994
Handwriting	0.963	0.848	0.999

95% Confidence Interval.

## Discussion

We assessed different ways to perform features of the TETRAS and found significant differences in scoring as a function of sometimes overlooked and unspecified variations in methodology. Similar tremor assessments are also used in other tremor scales [5].

Although neither a higher nor a lower mean score is “correct” we see advantages in using the method that generates a higher score, as we feel there is more likelihood of a floor effect compared to a ceiling effect with this scale, especially when used to assess treatment response.

In general, our results support assessing left and right arms individually, allows for photographs or writing samples to be rated without necessarily watching the subject perform these, and encourages cursive writing as opposed to script if possible, all of which are currently part of the TETRAS instructions. Our results contradict current TETRAS instructions by supported heal to shin testing done while sitting compared to supine. Our results also confirm excellent interrater reliability in arm assessments, but only moderate to good reliability in leg assessments. This is also consistent with previous data [2,3,4].

A few specific features warrant further comment. The greatest numeric difference between methods was seen in concurrent vs unilateral wing-beating tremor. It was hypothesized that concurrent bilateral assessments might actually increase tremor in the less severe hand via mechanical overflow, but in fact the less severe tremor side was rated higher when done individually, possibly owing to the rater subconsciously comparing the two arms when done concurrently, and lowered the less severe arm score.

It was also proposed that writing and spiral scores might be higher when the subject was viewed writing them, as some subjects showed marked arm tremor but manage to compensate to draw a relatively good figure. In fact, still photographs of both writing samples were slightly higher, although the correlation was very high (r = 0.97).

Handwriting may be especially patient specific. Although 19/24 subjects had higher scores with cursive (*X*^2^ < 0.05), 4 subjects had greater than a mean 0.5 point higher mean score with script, so this needs to be done consistently. Since a growing number of people do not learn cursive, script may eventually be the standard writing assessment at some point.

Heal to shin scores are usually low in ET but sitting heal to shin tremor scores were greater than supine scores. Heal to shin is traditionally done supine in ataxia scales but since this does add time and effort to the TETRAS scale, this data argues for a sitting heal to shin, Currently the total TETRAS score incorporates only the greater of the postural leg score or heal to shin score. In our analysis evaluating postural leg tremor vs supine heal to shin, the postural leg tremor is usually scored higher [1], arguing that heal to shin testing could be abandoned entirely.

## Additional Files

The additional files for this article can be found as follows:

10.5334/tohm.64.s1 Supplemental database file 1. Statistical analysis of datapoint.

10.5334/tohm.64.s2 Supplemental database file 2. Individual data scores.
